# Does sleep protect memories against interference? A failure to replicate

**DOI:** 10.1371/journal.pone.0220419

**Published:** 2020-02-13

**Authors:** Carrie Bailes, Mary Caldwell, Erin J. Wamsley, Matthew A. Tucker

**Affiliations:** 1 University of South Carolina School of Medicine Greenville, Greenville, South Carolina; 2 Department of Psychology and Program in Neuroscience, Furman University, Greenville, South Carolina; University of Hull, UNITED KINGDOM

## Abstract

Across a broad spectrum of memory tasks, retention is superior following a night of sleep compared to a day of wake. However, this result alone does not clarify whether sleep merely slows the forgetting that would otherwise occur as a result of information processing during wakefulness, or whether sleep actually consolidates memories, protecting them from subsequent retroactive interference. Two influential studies suggested that sleep protects memories against the subsequent retroactive interference that occurs when participants learn new yet overlapping information (interference learning). In these studies, interference learning was much less detrimental to memory following a night of sleep compared to a day of wakefulness, an indication that sleep supports this important aspect of memory consolidation. In the current replication study, we repeated the protocol of and, additionally, we examined the impact of intrinsic motivation on performance in sleep and wake participants. We were unable to replicate the finding that sleep protects memories against retroactive interference, with the detrimental effects of interference learning being essentially the same in wake and sleep participants. We also found that while intrinsic motivation benefitted task acquisition it was not a modulator of sleep-wake differences in memory processing. Although we cannot accept the null hypothesis that sleep has no role to play in reducing the negative impact of interference, the findings draw into question prior evidence for sleep’s role in protecting memories against interference. Moreover, the current study highlights the importance of replicating key findings in the study of sleep’s impact on memory processing before drawing strong conclusions that set the direction of future research.

## Introduction

Retention of newly learned declarative memories (e.g., fact-based information) improves following an interval filled with sleep as opposed to wakefulness [1–3]. However, there is considerable debate about how to characterize sleep’s role in memory processing, and specifically, whether sleep benefits memory primarily due to *active* or *passive* mechanisms [4].

A key feature of memory “consolidation”, as classically defined, is the development of increasing resistance to interference over time [5,6]. If sleep serves to stabilize newly acquired memories in this way, then information should not just be better remembered following sleep (compared to wake), but should also be more resistant to the interfering effects of new learning that occur after sleep has had a chance to consolidate these memories. It may be, however, that sleep does not stabilize memories, but merely slows the process of forgetting that would occur more rapid during wakefulness when the brain is more active and engaged in information processing [7]. In this case, retention of information would still be superior to wake following sleep (without interference), but sleep would not protect the memory against interference (e.g., learning information that is similar to the originally-learned information), leading to a similar rate of subsequent forgetting regardless of whether sleep or wake preceded the interference [4].

The current replication is based on two influential studies that support the hypothesis that sleep protects memory from subsequent interference, reporting that interference was less disruptive to memory when sleep, as opposed to wake, preceded interference learning [8,9].

### Sleep and declarative memory processing

Although the term “consolidation” today has multiple meanings, memory consolidation was originally defined as the process by which an initial labile memory trace becomes increasingly resistant to retroactive interference over time [5,10]. According to this view, if memories are consolidated during sleep, then interfering information should have less of a negative impact on post-sleep memory retention, compared to a much stronger negative effect on post-wake retention. From a neurobiological perspective, a number of candidate aspects of sleep have been suggested to play an active role in memory consolidation. For example, studies have observed correlations between time spent in stage 2 sleep or slow wave sleep (SWS) and declarative memory retention [11–13], suggesting that sleep stage-specific neural activity during these particular stages may contribute to memory consolidation. Sleep-specific oscillations, such as sleep spindles [14,15] and other neuronal oscillations that occur during sleep, such as hippocampal ripples [16], have also been correlated with memory processing. While these studies are compelling, it should be noted that experimental manipulations of these aspects of sleep are still limited in number, and alternative hypotheses exist. Indeed, some suggest that sleep, like other physiological states (e.g., those induced by alcohol or benzodiazepine use, for example), allows consolidation to occur simply because new learning is dramatically reduced during these times [7]. This line of reasoning suggests that sleep may be one of many possible states during which memory consolidation can occur, though it may be the most opportune time [17]. Thus, even if active processes of memory consolidation are occurring during sleep, this may not necessarily be due to specific properties of sleep or sleep-specific neuronal events.

### Sleep, memory, and interference

A small number of influential studies have suggested that sleep reduces the negative impact of retroactive interference on memory performance. One study, using an interference paradigm similar to the one employed in the current study, examined performance in older (ages 50–79) and younger (ages 18–30) adults using a visuospatial declarative memory task, finding that sleep (relative to daytime wakefulness) reduced the impact of interference on memory in younger participants and in high-performing older participants [18]. Analogously, a study examining sleep and interference in songbirds using an auditory classification task found that the detrimental effects of learning competing auditory classifications were reduced following sleep, but not wake [19].

Other studies have examined how specific sleep stages may contribute to the reduction of interference effects. One study, using a 1.25-hr training-retest interval filled with wake, a 10-minute nap (without SWS), or a 60-minute nap (with SWS), found that the effects of interference were reduced in those who obtained SWS during the 60min nap, compared to those taking the 10min nap [20]. However, the effects of interference in the nap groups did not differ significantly from the effect of interference in the waking control group. In a similar nap study using word pair learning, participants who obtained SWS during a nap experienced less forgetting due to interference learning than those who did not obtain SWS [21]. These last two studies have both been interpreted to suggest that SWS, specifically, may promote the strengthening of memories against subsequent interference.

However, two studies by Ellenbogen, et al. [8,9] have been by far the most cited and most influential evidence for sleep’s proposed role in protecting against interference. These studies form the basis for the present replication. Employing a classic interference learning protocol developed by Barnes and Underwood [22], these two studies had participants learn a list of word pairs (A-B pairs) in the evening (prior to a night of sleep) or the morning (prior to a day of wake), followed by an immediate test of the word pairs at the end of the learning session [8,9]. In the protocol followed by Ellenbogen, et al. (2009) [9], which we replicate in the current study, participants returned 12 hours later to be retested on some of the word pairs. Not surprisingly, they found that those who had slept retained more word pairs than those who remained awake. Participants then underwent interference training for a subset of the originally-learned word pairs (A-C word pairs), followed by a test of the original B associates and the newly learned C associates. Interference training lead to a dramatic forgetting of the originally learned words in the wake participants, compared to those who slept.

While there has been no previous direct replication of the Ellenbogen studies, one study by Sheth, Varghese, and Truong (2012) partially replicated their methods. Using the same paired associates task, and same number of word pairs (20), the authors found that sleep benefitted recall of the original B words after participants had been exposed to interference training, and when they were trained to get the word pairs correct 3x or 1x at training (Experiments 1 and 2) [23]. However, there were a few limitations in that study that may preclude a direct comparison to Ellenbogen, et al. (2009). First, in Experiments 1 and 2 participants were not tested on the 20 word pairs at the end of the training session, nor were they tested at the beginning of the retest session, prior to interference learning, as was done in Ellenbogen, et al. (2009) and the current study. The lack of a pre-interference test precludes replication of the critical Sleep/Wake x Interference/No-Interference interaction that could reveal the magnitude of sleep’s hypothesized protective effect. In Experiment 4, when these additional tests were included, interference did not negatively impact Sleep participants more than Wake participants. It is possible that participants overlearned the word pairs at training, which may have masked Sleep/Wake differences that would have emerged with a less well-instantiated memory trace at training. It should also be noted that the sample sizes used in the Sheth et al. study were smaller than in the Ellenbogen study, with n = ~10 in each cell.

Taken together, these studies suggested to many that sleep might support a key aspect of consolidation: resistance to interference. However, not all studies have uniformly supported this hypothesis, and a direct replication of Ellenbogen et al.’s original studies has not yet been attempted.

### The present study

The current study was designed to detect the protective effect of sleep against subsequent interference reported by Ellenbogen et al. (2009) [9]. As reported in that study, we expected that interference learning would lead to a greater sleep (vs. wake) memory benefit than that observed when no interference learning was undertaken by participants (i.e., a significant sleep/wake x no-interference/interference interaction). Alternative patterns of interaction (i.e., a *smaller* effect of sleep for no-interference, relative to interference learning) would not be considered a successful replication. We also anticipated replicating the main effects of sleep benefitting memory performance, and retroactive interference harming memory performance.

In this study we also examined the impact of intrinsic motivation on memory performance. Past research has shown that providing a monetary reward (extrinsic reward) for performing better on a task (e.g., a simple typing task) led to greater task improvement following sleep compared to wake [24]. However, one study from our lab was unable to replicate this effect using a declarative picture pairs task [25]. More recently, our lab has examined *intrinsic* motivation (the desire to perform a task well for its own sake) as a potential modulator of the sleep-wake differences in memory. One study from our lab showed that intrinsic motivation correlated with better task acquisition *and* consolidation (change in performance from training to retest), but was not associated with better performance in the sleep (v. wake) participants [26]. As in that study, we analyzed four items from the Intrinsic Motivation Inventory [27,28] and their relationship to memory performance following sleep and wake. We hypothesized that greater intrinsic motivation would be associated with better declarative memory at training and better retention across the 12-hr training-retest interval, but that intrinsic motivation would not provide an added memory benefit to those who slept as opposed to stayed awake during the retention interval.

## Methods

### Participants

Participants were 97 university students (age: 23.2±1.9yrs, range: 18–28, 59 female). Sleep (n = 51) and Wake (n = 46) participants were asked to sleep well the night before the study and not to exceed their usual daily caffeine intake. Of the 108 participants who completed the study, eleven were excluded from analysis for 1) taking stimulant and/or antidepressant medications (n = 9) or 2) reporting that they obtained >2hrs less than their typical total sleep time the night before the study (n = 2). While we attempted to match our selection criteria to those employed in the Ellenbogen, et al. (2009) study, there were a few differences between our study and the original. For example, participants in the original study were excluded if they had a habitual bed time after 2am and habitually sleep less than 6hrs per night. The Ellenbogen, et al. (2009) study also only enrolled right-handed participants, and participants were excluded if they had Epworth Sleepiness Scale scores >10. In the current study handedness was not assessed, but participants did report habitual bed times of 2am or earlier and total sleep times of 5hrs or greater, with only n = 4 obtaining between 5-6hrs of sleep. All participants were native English speakers except for three who had >14 years of English language experience. In the current study we did not require that participants report Epworth Sleepiness Scale scores <10 (n = 21 reported scores >10), but we found no correlation between ESS score and training/retest recall (all p-values >0.17). We also did not screen for psychiatric, sleep, or neurological disorders, but we did exclude participants who were taking stimulants and/or antidepressants, and no participants reported taking medications known to affect sleep and cognition, including antipsychotic medications, antihistamines, or sleep aids (e.g., Ambien or Nyquil). All participants provided informed consent to participate in the study. The study was approved by the Prisma Health System IRB and the Furman University IRB.

### Paired associates task

**Fig 1:**
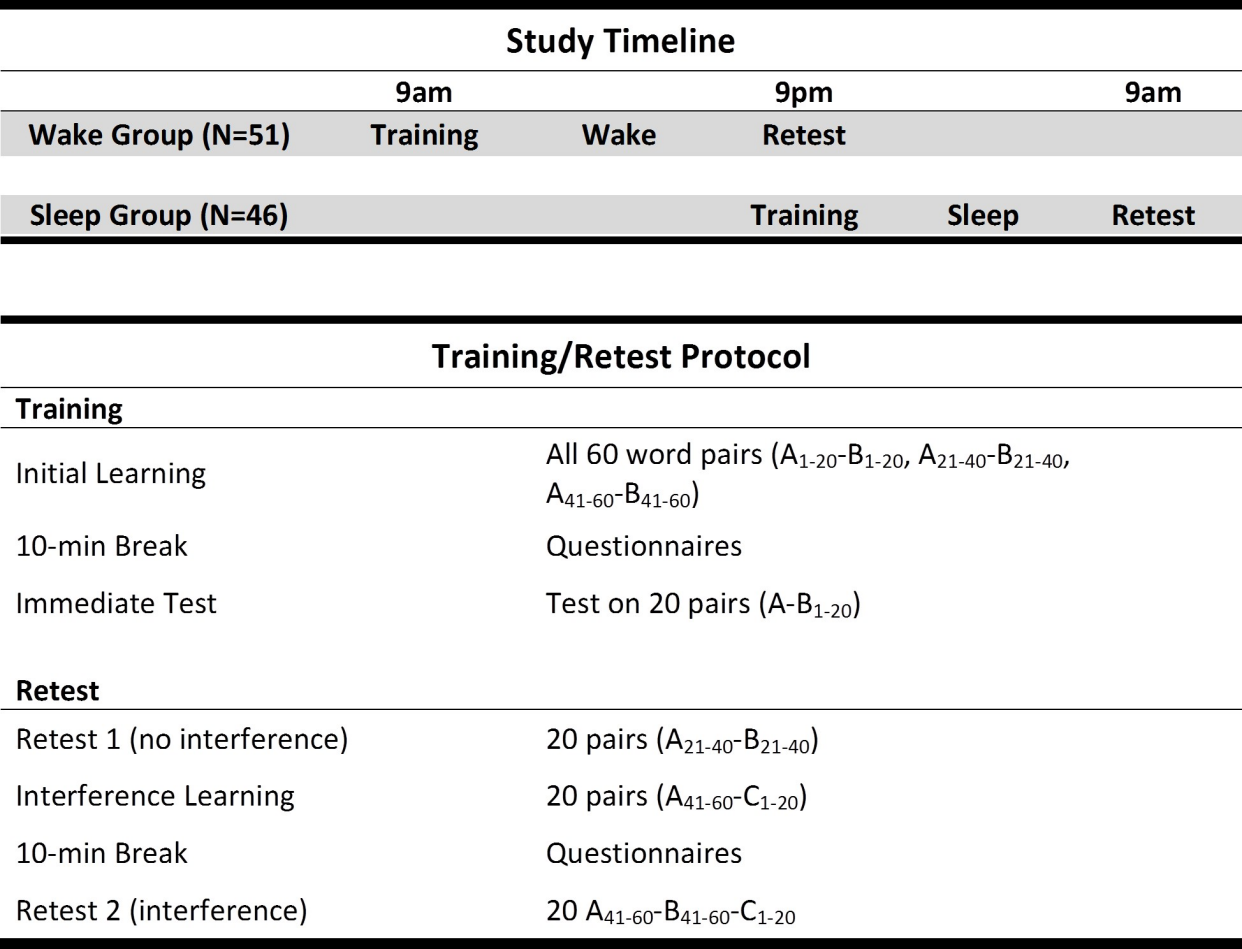
Study timeline and protocol. The top part shows the timeline for the Sleep and Wake participants from training to retest. The bottom part describes the word pairs training and retest protocol.

Here we used the paired associates task from Ellenbogen et al. (2009) [9], designed to provide a within-subjects manipulation of interference. This represented a methodological improvement over the Ellenbogen et al. (2006) study [8], in which sleep/wake condition and interference condition were treated as between-subjects variables, decreasing the statistical power of the design. The word pairs used in the current study were the same ones used in Ellenbogen et al. (2009). In their study they randomly selected two-syllable words from the Toronto Word Pool [29], which were used to create three lists of 20 word pairs (A_1-20_-B_1-20_, A_21-40_-B_21-40_, A_41-60_-B_41-60_) and a fourth list used during the interference learning phase (A_41-60_-C_1-20_) (see Fig 1). Word pairs were “matched for imageability, frequency of use, and concreteness” [9]. Examples of word pairs: A-B pair: CARPET-BUBBLE / A-C pair: CARPET-PAPER (same cue word, different target word). The paired associates task was implemented using OpenSesame stimulus presentation software [30]. Assignment of words to list was counterbalanced using three orders (list 1, 2, 3; list 2, 3, 1; list 3, 1, 2), ensuring that each word list was equally represented in each serial position. At training, N = 14 Sleep/n = 19 Wake participants were presented list #1 first, n = 17 Sleep/n = 12 Wake participants were presented list #2 first, and n = 20 Sleep/n = 15 Wake participants were presented list #3 first.

During the initial training session in the morning (9am) or evening (9pm), all 60 word pairs were presented for 7s each. Participants were instructed to memorize the pairs but were not given specific strategies about how to memorize them. Following presentation of the word pairs, participants were then quizzed on all 60 pairs, during which they were presented the first word of the pair (A) and were instructed to enter the word that was paired with it (B) in a text box next to the cue word. If participants correctly typed the B word, the pair would not appear again during quizzing. If a response was not correct, the correct pairing would appear on the screen and the item would appear again for another attempt after a random interval. The quizzing phase was complete once all of the A-B pairs were entered correctly one time. There was then a 10-minute break period, during which participants completed paper-pencil study forms. After the 10-minute break, participants were tested on 20 of the 60 word pairs (e.g., List 1: A_1-20_-B_1-20_) as a measure of immediate memory (Immediate Test). During this test no feedback was provided after a response was entered.

Twelve hours later, after a night of sleep (9am) or a day of wake (9pm), participants returned for the retest session. During the first part of this session participants were tested on a different set of 20 word pairs from the original set of 60 (e.g, A_21-40_-B_21-40_; Retest 1), a test of memory for word pairs uninfluenced by interference learning. Following this memory test, participants completed the interference learning phase by learning a new set of 20 word pairs that overlapped with the remaining 20 word pairs from the original learning session (e.g., A_41-60_-B_41-60_). This A-C list contained the same A words that were learned originally, paired with 20 new C words (A_41-60_-C_1-20_). The 20 A-C pairs (A_41-60_-C_1-20_) were learned in the same way as the original 60 word pairs, with presentation of the word pairs followed by quizzing with feedback until all pairs were correctly answered one time. There was a 10-minute break following A-C interference learning, during which participants completed the remaining study forms. After the break participants were tested on both the B_41-60_ and C_1-20_ words that had been paired with A_41-60_ (post-interference Retest 2). The A word for each pair was presented and participants attempted to enter both the B word (from the training session) and C word (from the interference session) that had been paired with it. The number of B and C words correct was used as the primary dependent variable. Items were counted as correct if the answer was misspelled but clearly referred to the answer (e.g., ‘buton’ for ‘button’; ‘blankets’ for ‘blanket’).

### Procedure

All sessions were conducted either in a lecture hall at the University of South Carolina School of Medicine Greenville (n = 87), or in a computer laboratory at nearby Furman University (n = 10). Participants either participated in a Wake group that completed the training session at 9am and the retest session at 9pm, or a Sleep group that trained at 9pm and retested the next morning at 9am (Fig 1). Upon arrival, participants signed the consent form, and then trained on the 60 word pairs. Following training, during the 10-minute break that preceded an Immediate Test on the A_1-20_-B_1-20_ word pairs, participants completed a set of forms that included a demographics form, a 3-day retrospective sleep log, alertness/sleepiness scales, and the Epworth Sleepiness Scale [31], a measure of the likelihood of falling asleep in eight situations (e.g., sitting and reading a book). The alertness/sleepiness scales consisted of two visual analog scales: “How would you describe your ability to concentrate right now?” and “How refreshed do you feel right now?”, and the Stanford Sleepiness Scale [32], which measures how alert/sleepy the participant feels at that moment. Following the immediate test on the A-B word pairs, participants were free to leave, and were instructed to limit caffeine intake and not to nap (Wake participants) prior to returning for the retest session. For the immediate test at the end of training, as well as the two tests during the retest session, participants were allowed as much time as needed to enter their answers. The duration of the training session was 30-40min.

When participants returned 12 hours later, they were retested on 20 of the items learned during the training session, items not tested at the end of the training session (e.g, A_21-40_-B_21-40_; Retest 1). This initial test was followed by interference learning of the A-C word pairs. During the 10-minute break that followed A-C interference learning, participants completed forms that included the alertness/sleepiness scales, a sleep or wake log that documented participant activities during the training-retest interval, and a ‘Task Perception Inventory’ that included four items from the from the Intrinsic Motivation Inventory (IMI) [27,28,33]: “I enjoyed doing the word pairs task very much” (Enjoyment), “I didn't try very hard to do well at the word pairs task” (Effort), “I think I am pretty good at the word pairs task” (Competence), and “I felt very tense while doing the word pairs task” (Tension). These questions were answered on a 7-point Likert scale: “Not at all true” to “Very True”. Participants then answered two questions on a visual analog scale (measured in mm): “How motivated were you to do well on the word pairs task?” (Motivation) and “How much did you think about the word pairs task before the retest session?” (Thinking About). After completing the final test (A_41-60_-B_41-60_-C_1-20_; Retest 2), the study was concluded and participants were paid for their participation. The duration of the retest session was 30-40min.

### Power analysis

To ensure the current replication study was well-powered to detect the large protective effect of sleep reported in Ellenbogen et al. (2009), effect size was calculated from the Ellenbogen et al. (2009) Sleep x Interference interaction effect, which was the primary statistic demonstrating that the effect of interference on memory depended on sleep/wake condition (η_p_^2^ (partial eta squared = 0.0985; Cohen (1988) [34] suggests η_p_^2^ benchmarks of 0.01 = “small”, 0.06 = “medium”, and 0.14 = “large”). This effect size and the confidence intervals displayed in Fig 3 were calculated in R, using the ci.pvaf function of the MBESS package (https://CRAN.R-project.org/package=MBESS).

Power analysis was conducted in G*Power [35] using the module for within/between ANOVA interaction effects, and the “as in SPSS” option for type of partial eta squared effect size. The sample used in this replication (N = 97), which was more than double the sample size of the original study [36], yields statistical power = 0.89 to detect the effect reported by Ellenbogen et al. (2009).

## Results

### Participant data

Means±SEMs are presented for the Sleep group first. Participants in the Sleep and Wake conditions reported similar sleep log and demographic data: Usual bedtime: 10:58pm±7.8min, 11:02pm±8.4min, p = 0.72; Usual total sleep time: 7.2±0.1hrs, 7.0±0.1, p = 0.21; Total sleep time the night before the study: 7.3±0.2hrs, 7.2±0.2hrs, p = 0.53; Epworth Sleepiness Scale score: 8.3±0.6, 7.7±0.6, p = 0.47. Self-report measures of sleepiness and alertness revealed that participants in the Wake group reported feeling more refreshed (39.7±3.9mm, 50.8±3.7, p = 0.04), marginally more alert (SSS: 3.0±0.2, 3.4±0.2, p = 0.09), but not better “able to concentrate” (58.4±3.6mm, 53.2±3.7, p = 0.33) than those in the Sleep group. While sleep and wake groups both reported feeling more alert, refreshed, and able to concentrate at retest compared to training, the differences favored sleep participants, who reported feeling more alert (SSS; p = 0.07), more able to concentrate (p = 0.05) and more refreshed (p = 0.001). Sleep subjects went to bed earlier than wake subjects the night before the study (11:22pm±8.4min, 12:23±10.2min, p<0.001) but, as noted above, total sleep times were comparable.

### Paired associates performance

*Training*: At training, Sleep and Wake groups performed similarly on the immediate test of B words (Percent correct: 82.1±2.7% (16.4 pairs), 83.8±2.6% (16.8), t_(95)_ = 0.46, p = 0.64; Trials to reach criterion: 152.0±9.1, 147.0±14.5, t_(95)_ = 0.30, p = 0.77).

**Fig 2:**
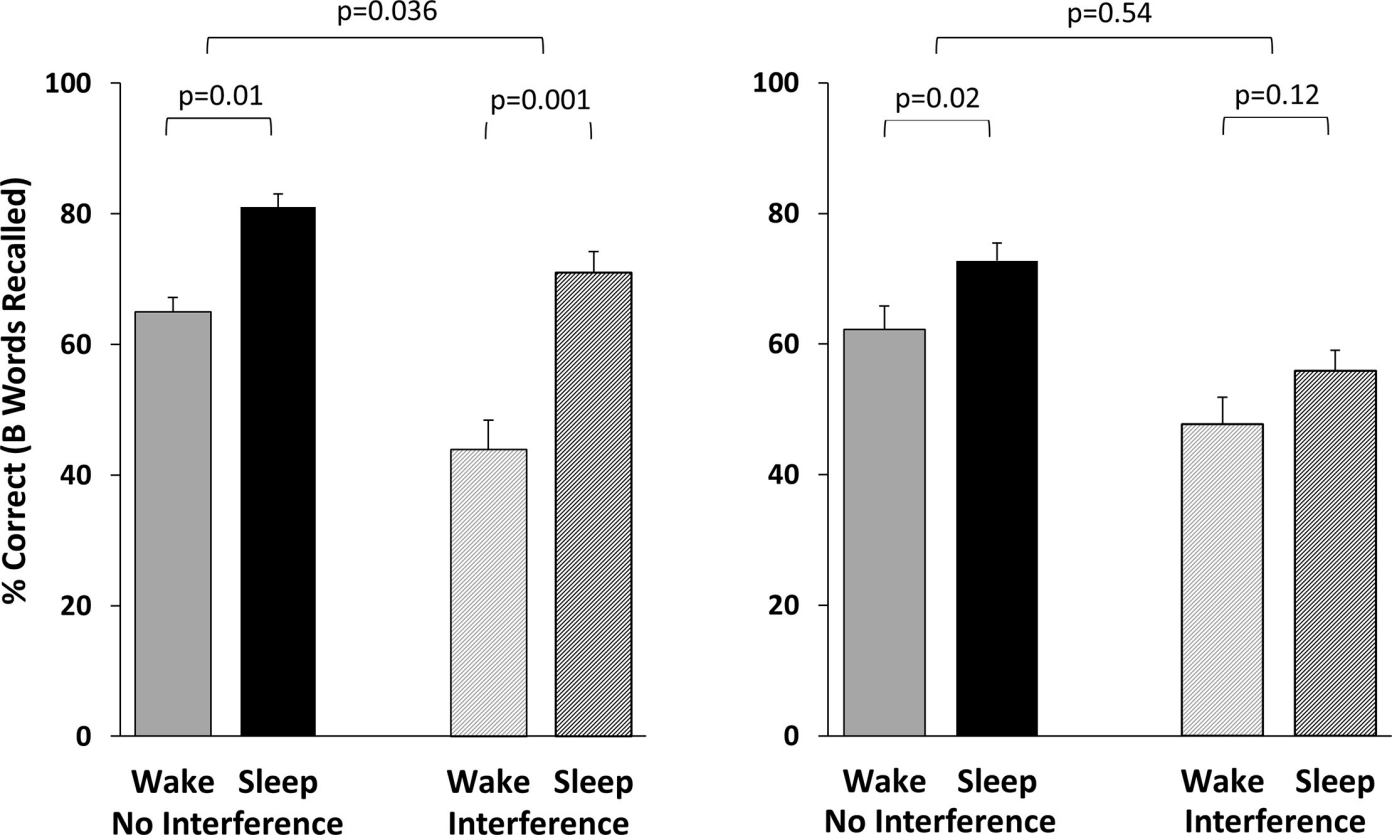
Paired associates results. Results from Ellenbogen et al. (2009) (Left) are compared to findings from the current study (Right). Results for “no interference” represent recall from the initial retest, prior to interference learning. “Interference” results represent recall of originally-learned B words following interference learning.

*Retest*: A 2x2 ANOVA was conducted to examine the effects of Sleep v. Wake (between subjects) and Interference v. No-Interference (within subjects) on memory for the B words at retest. As expected, there was a significant main effect of Sleep v. Wake, with memory for B words being superior in the Sleep group (F_(1,95)_ = 4.44, p = 0.038, η_p_^2^ = 0.04). There was also a significant main effect of Interference v. No-Interference, with memory being superior for the No-Interference pairs (F_(1,95)_ = 70.61, p < .0001, η_p_^2^ = 0.43). However, crucially, there was no Sleep x Interference interaction effect, with Sleep participant performance dropping 16.9±2.3% from Retest 1 (before interference training) to Retest 2 (after interference training), and Wake participants dropping 14.6±3.0% (F_(1,95)_ = 0.38, p = 0.54, η_p_^2^ = 0.004), indicating that the effect of sleep on memory did not differ between Interference v. No-Interference pairs (Fig 2). Order of list presentation did not affect recall at immediate test at training (Order 1: 16.1±0.6, Order 2: 16.2±0.8, Order 3: 17.3±0.6, F_(2,94)_ = 1.10, p = 0.34), and adding trials to criterion or number correct at immediate test (at the end of the training session) as covariates in the ANOVA had no impact on the results.

*Performance at Each Time Point*: Retest 1 –Prior to interference training: Sleep participants recalled more B words than Wake participants: 72.7±2.8%, 62.3±3.5%, t_(95)_ = 2.35, p = 0.02, d = 0.33. Retest 2 –After interference learning: The Sleep-Wake difference for post-interference memory for B words did not reach significance: 55.9±3.1%, 47.7±4.1, t_(95)_ = 1.59, p = 0.12, d = .23. Interference (A-C) training: Sleep and Wake groups recalled a similar percentage of interference (AC) word pairs: 78.6±3.1, 76.5±3.6, t_(95)_ = 0.45, p = 0.65, d = 0.06). The number of trials required to reach criterion for A-C training was also similar between groups (36.6±2.0, 33.9±1.9, t_(95)_ = 0.97, p = 0.34, d = 0.14).

### Intrinsic motivation

Table 1 shows the correlation of memory performance with measures of task perception (items from the Intrinsic Motivation Inventory) and the ‘Motivate’ and ‘Thinking About’ visual analog scales. We found that three items in particular (Enjoyment, Competence, and Tension) correlated with performance (correct items) at training, Retest 1 (pre-interference learning), and Retest 2 (post-interference learning). The VAS question regarding how motivated participants were to do well on the task also correlated with recall at these three time points. However, there were no significant correlations between any of these questionnaire measures and change in performance from training to retest. Fisher’s Z tests revealed no significant differences between correlations for Sleep vs Wake participants.

**Table 1:** Correlations between indices of intrinsic motivation from the Intrinsic Motivation Inventory, training performance and change in performance from training to retest.

		Training (Immediate test)		Retest1 (No interference)		Retest2 (Interference)	
		r	p	r	p	r	p
Intrinsic Motivation Inventory	Enjoyment	.42	**< .001**	.42	**< .001**	.36	**< .001**
	Effort	.14	.17	.14	.17	.05	.63
	Competence	.55	**< .001**	.49	**< .001**	.39	**< .001**
	Tension	-.40	**< .001**	-.26	**.01**	-.19	.06
	Total Score	.53	**< .001**	.45	**< .001**	.35	**< .001**
Visual Analog Scales	Motivation	.31	**.002**	.23	**.02**	.23	**.03**
	Thinking About	-.05	.65	.14	.17	.10	.35

Retest 1: First test of the retest session, prior to interference learning (A_21-40_-B_21-40_): Retest 2: Post-interference learning retest on the original B words (A_41-60_-B_41-60_). Significance threshold after Bonferroni correction for multiple correlations (p<0.0035 for IMI items and p<0.0085) [37]. Bold: significant correlations, p<0.05.

## Discussion

The studies by Ellenbogen et al. [8,9] that form the basis for the current replication were important because they suggested that sleep does more than passively protect memories from interference. Specifically, these studies suggested that sleep stabilizes memories over the training-retest interval, such that interference learning after a night of sleep has a much smaller impact on memory than after a day of wakefulness. According to the authors, this central finding “demonstrates the active role of sleep in consolidating memory” [9].

In the present study, as in the Ellenbogen studies, we found that, prior to interference training, those who slept had better memory for the word pairs than those who were awake during the 12hr interval. However, we did not replicate Ellenbogen et al.’s observation of a large effect of sleep in protecting memories against interference learning. Instead, we observed that the Sleep and Wake groups both experienced a significant and very similar retroactive interference effect.

One reason for the discrepancy between our findings and those of Ellenbogen et al. [9] may relate to differences in testing protocol. In their first study [8], participants were required to get each word pair correct twice during training, which resulted in a very high rate of memory performance at retest in the sleep group (with sleep participants getting 94% of items correct at Retest 1). This suggested a ceiling effect that may have artificially attenuated the effect of sleep on recall of non-interference word pairs (Retest 1, where performance levels were highest), thus creating a spurious interaction between sleep-wake condition and interference condition. In their 2009 follow-up study [9], this ceiling effect was no longer apparent–although some participants were again required to answer each item correctly twice, while others were required to answer them correctly only once. In that 2009 study, the sleep-wake x interference-no interference interaction was slightly diminished compared to their original 2006 finding, though still statistically significant. In contrast, in the current study, all participants were required to get each item correct only once, which kept the training protocol the same for all participants, and reduced the risk of ceiling effects at initial retest (Retest 1).

Given the likelihood that participants were over-trained in both the Sheth, et al. (2012) [23] (3x correct at training) and the Ellenbogen et al. (2006) study (2x correct), having participants answer items correctly only one time in the present study should have been sufficient to replicate the interference effect observed in Ellenbogen, et al. (2009), without encountering ceiling effects. Thus, it is possible that the apparent ability of sleep to protect against interference in Ellenbogen et al.’s original paper [8] was the spurious result of ceiling effects to which our current data were not susceptible.

One difference in procedure between the current replication and the Ellenbogen et al. (2009) study was that our participants completed forms during the 10-minute breaks at training and retest, whereas non-verbal tasks were performed during the breaks in the Ellenbogen et al. 2009 study. Reading the forms and describing their experience performing the tasks may have produced added interference that would not have been produced in the Ellenbogen study. We cannot rule out the possibility that this additional interference during the breaks may have influenced our current results.

**Fig 3:**
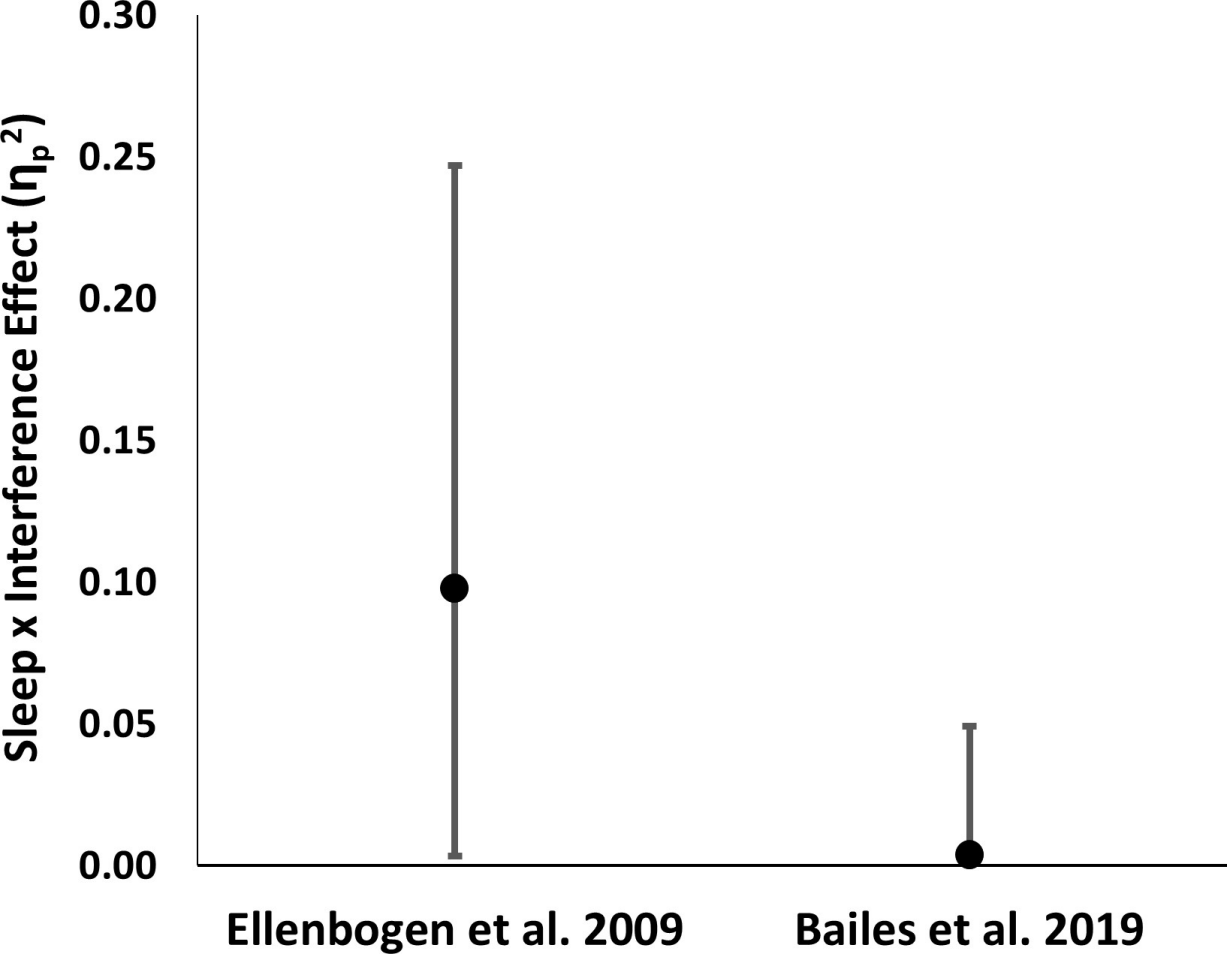
Effect size comparison. Points represent the size of the Sleep x Interference interaction effect as reported by Ellenbogen et al. (2009), and in the current study (partial eta squared). Error bars represent the 90% confidence interval on this effect. The Ellenbogen et al. 2009 effect falls well outside the 90% confidence interval of the current, more precise effect size estimate.

The failure to detect the large-sized effect reported by Ellenbogen et al. is not likely due to a lack of statistical power. The sample size of our study was more than 2x as large at that used in Ellenbogen et al. (2009), and thus we were powered to replicate their effect at 0.90. The observed effect size for our sleep/wake x no-interference/interference interaction was near-zero (partial eta squared = 0.003), and the Ellenbogen et al. (2009) effect (partial eta squared = 0.0985) fell outside the 90% confidence interval of our effect (Fig 3).

Importantly, although our observations are consistent with a small protective effect of sleep against interference, Ellenbogen’s 2009 study would have been powered at just 0.27 to detect even the largest effect consistent with our 90% confidence interval. Thus, while Ellenbogen et al.’s (2009) effect arguably could have been an overestimate of a true, but small effect, it is unlikely that they could have detected an effect small enough to be consistent with our current observations. The alternative possibility is that Ellenbogen et al.’s 2009 effect was a false positive. As a result, our confidence in the evidentiary value of the Ellenbogen et al. studies has been reduced.

We have long viewed the Ellenbogen et al. studies as some of the most important positive evidence for a protective role of sleep in mitigating the negative impact of subsequent interference. While other studies reviewed here have also supported this hypothesis to a degree, Ellenbogen’s core finding that post-training sleep reduces the effects of subsequent interference has never been directly replicated. To be clear, our current study cannot rule out the existence of a small effect, as small effect sizes are well within the 90% confidence interval of our observation. However, we note that all prior published reports of sleep’s hypothesized role in protecting against interference have been in the medium-to-large range. Thus, a potential small-sized effect of sleep protecting against interference remains a hypothetical. For these reasons, we remain agnostic on the question of whether sleep protects against retroactive interference. Although this may be the case, there is currently little evidence to support this conclusion.

A second aim of this study was to examine intrinsic motivation and its relationship to measures of memory performance. A previous study from our lab found that providing participants an extrinsic reward for performance ($1 per correct answer) resulted in better recall of visual paired associates (picture pairs) compared to no reward, but that this reward effect was not different between sleep and wake participants [25]. We have also shown that increased intrinsic motivation (the desire to do well even in the absence of extrinsic rewards), measured by responses to items from the Intrinsic Motivation Inventory [27] used in the present study, correlated with better acquisition of visual paired associates (picture-object pairs) at training, and also with change in performance from training to retest (retention) [26]. In the present study, we observed a similar positive correlation between intrinsic motivation and training and retest performance. However, motivation did not correlate with memory consolidation (change in performance from training to retest), nor did intrinsic motivation favor performance in the sleep (v. wake) participants.

This difference in results may be attributable to the nature of the task. While both tasks are declarative memory tasks, the visual paired associate stimuli used in our past studies are pairs of images, as opposed to the word pairs used in the current study. It is possible that the sensory modality employed for acquiring the stimuli, as well as possible differences in task difficulty, could account for this difference in the two sets of findings. It should also be noted that in the current study, as with most studies on this topic, we paid participants for their participation in the study, which is a form of extrinsic reward, even though not tied to performance. We cannot rule out the possibility that the inclusion of such a payment may have impacted participants’ intrinsic motivation ratings. Alternatively, we acknowledge the current study’s lack of a motivation effect on memory change over time and sleep could be the result of Type II error. Further studies with larger sample sizes would be necessary to truly rule out an effect of motivation on memory consolidation in this paradigm. Together, these findings point to the potential importance of intrinsic motivation for optimizing learning (and memory consolidation for some tasks (e.g., visual paired associates)), but also suggest that intrinsic motivation may not be an important modulator of sleep-wake differences in memory.

While the studies by Ellenbogen et al. (2006, 2009) appeared to provide compelling evidence that sleep is actively stabilizing memories against interference, our findings draw this now commonly accepted finding into question. Although the current study is only one within a small literature describing sleep’s ability to reduce the impact of post-sleep interference [18–21], as reviewed above, at least two other prior studies have provided only equivocal support for sleep’s purported role in stabilizing memory against interference [20,23]. Thus, the current study suggests that further research is warranted before drawing strong conclusions about sleep’s role in stabilizing memories against subsequent interference. This study also highlights the importance of independent replication of seminal findings in the field, and suggests that challenges remain as we continue to define the specific nature of sleep’s role in memory processing.

## Supporting information

S1 Data (SAV)
